# Global health partnership for student peer-to-peer psychiatry e-learning: Lessons learned

**DOI:** 10.1186/s12992-016-0221-5

**Published:** 2016-12-03

**Authors:** Roxanne C. Keynejad

**Affiliations:** 1 Centre for Global Health, King’s College London, Weston Education Centre, Cutcombe Road, London, SE5 9RJ UK; 2 Institute of Psychiatry, Psychology & Neuroscience, King’s College, London, UK

**Keywords:** Global mental health, Peer-to-peer, E-learning, Psychiatry, Global health, Health partnerships, Twinning, Internet, Medical education, Low income countries

## Abstract

**Background:**

Global ‘twinning’ relationships between healthcare organizations and institutions in low and high-resource settings have created growing opportunities for e-health partnerships which capitalize upon expanding information technology resources worldwide. E-learning approaches to medical education are increasingly popular but remain under-investigated, whilst a new emphasis on global health teaching has coincided with university budget cuts in many high income countries.

**Results:**

King’s Somaliland Partnership (KSP) is a paired institutional partnership health link, supported by Tropical Health and Education Trust (THET), which works to strengthen the healthcare system and improve access to care through mutual exchange of skills, knowledge and experience between Somaliland and King’s Health Partners, UK. *Aqoon*, meaning *knowledge* in Somali, is a peer-to-peer global mental health e-learning partnership between medical students at King’s College London (KCL) and Hargeisa and Amoud Universities, Somaliland. It aims to extend the benefits of KSP’s cross-cultural and global mental health education work to medical students and has reported positive results, including improved attitudes towards psychiatry in Somaliland students.

**Conclusions:**

The process of devising, piloting, evaluating, refining, implementing, re-evaluating and again refining the Aqoon model has identified important barriers to successful partnership. This article describes lessons learned during this process, sharing principles and recommendations for readers wishing to expand their own global health link beyond qualified clinicians, to the healthcare professionals of the future.

**Electronic supplementary material:**

The online version of this article (doi:10.1186/s12992-016-0221-5) contains supplementary material, which is available to authorized users.

## Background

Long-term global ‘twinning’ relationships between healthcare organizations and institutions in low and high-resource settings are attracting growing attention [1]. Over 15 years, partnerships of this kind contributed to Millennium Development Goal 8: ‘to develop a global partnership for development’ [2] in the broadest sense, by sharing knowledge, skills and experience, to strengthen global healthcare. This international objective was expanded in Sustainable Development Goal 17, which explicitly advocates that we ‘strengthen the means of implementation and revitalize the global partnership for sustainable development’ [3]. Outside of face-to-face learning and knowledge exchange, these links additionally create growing opportunities for e-health partnerships which capitalize upon expanding information technology resources worldwide.

Authorities representing AMPATH (Academic Model Providing Access to Healthcare), Earth Institute, Columbia University, Brigham and Women’s Hospital and the World Health Organization have advocated for ‘dissemination of details of successful partnerships… in which e-health partners share their methods and provide mutual support’ [4]. They identified ‘cultural differences and geographic distances’, ‘miscommunication and misunderstanding’, ‘maintaining funding and momentum for initiatives’ and ‘lack of consistent, basic services such as electricity and internet connectivity’ as the four central obstacles to successful electronic partnerships.

Peer teaching has well-documented benefits, including increased knowledge, clinical and communication skills, time management, confidence and taking responsibility [5]. Online peer-to-peer study exploiting e-learning technologies offers a more diverse medical education, with access to worldwide resources [6]. Where students have the same learning objectives, a single web-based environment is effective for cohorts from contrasting cultures, provided they are fluent in the same language [7]. However, the variation within so-called web-based learning is extensive, with correspondingly diverse outcomes [8].

E-learning methods are increasingly popular but remain under-investigated. Combining more traditional teaching methods with computer-based learning overcomes the problem of context-dependence when only one modality is used [9]. A meta-analysis of computer-assisted learning for healthcare professionals found improved examination scores [10] and studies support an interactive, student-c**e**ntered approach [11]. Interactive approaches also generate greater learning than non-interactive web-based education, for both trainee doctors and medical students [12].

There is growing emphasis on global health in medical education, encapsulated by the inclusion of the learning outcome: ‘discuss from a global perspective the determinants of health and disease and variations in health care delivery and medical practice’ in *Tomorrow’s Doctors* [13]. Recessionary university budget cuts [14] mean innovative, cost-effective technologies stimulating interest in global health are increasingly important to educators and clinicians worldwide.

Somaliland is a self-declared independent state in northern Somalia. Its estimated population is 2–3.5 million [15], with an annual health budget of $750, 000. There are only three public inpatient psychiatric units in the country and no psychiatrists working in the public sector. The 110-bed Hargeisa Group Hospital is staffed by auxiliary nurses and had no medical input until recently [16], resulting in a large unmet regional burden of mental illness [17, 18].

King’s Somaliland Partnership (KSP) is a paired institutional partnership health link supported by Tropical Health and Education Trust (THET) [19], which works to strengthen the healthcare system and improve access to care through mutual exchange of skills, knowledge and experience between Somaliland and King’s Health Partners, UK [20]. This includes teaching and examination support from UK psychiatrists to medical students who would not otherwise receive formal mental health training. KSP medical student psychiatry teaching is associated with increased knowledge and improved attitudes towards mental health [21]. The sustainability of teaching initiatives is preserved through a KSP mental health representative based in Somaliland [22].

MedicineAfrica is a telemedicine portal based on a social network structure which facilitates online case-based tutorials in real time. It has been used for instant messaging distance teaching in Sierra Leone and Somaliland, where partners received reciprocal educational benefit [23]. Its goal is to connect, train, support and empower isolated members of the global health workforce using innovative technology and responsible programs. It offers a system whereby a network of clinical workers may upload contextually relevant health information including anonymized clinical cases for education and mentoring.

The expertise and experience of KHP shared through KSP, via MedicineAfrica created an ideal opportunity to find out whether global health links could be usefully expanded to involve medical students in both low and high-income settings. This article describes the process of devising, piloting, evaluating and refining a model of peer-to-peer e-learning through KSP. It describes lessons learned during this process, barriers to successful partnership, principles and recommendations for sharing the benefits of global health links with students, trainees and other interested parties.

## Methods

*Aqoon*, meaning *knowledge* in Somali, was the name chosen for a novel peer-to-peer global mental health e-learning partnership between medical students at King’s College London (KCL) and Hargeisa and Amoud Universities, Somaliland. Aqoon forms part of the KSP MedicineAfrica evaluation, with ethical approval from KCL Ethics Committee. Its purpose is to extend the education provided through KSP to medical students. Its aims are:

- ▪ To facilitate cross-cultural peer-to-peer global mental health education links between pairs of students in London and Somaliland.
- ▪ To create opportunities for mutual global health learning through collaborative discussion of understandings of mental illness and approaches to care and help-seeking.
- ▪ To challenge stigma towards people with mental illness and towards psychiatry.
- ▪ To stimulate students’ interest in global health, mental health and to facilitate exploration of the relationship between the two through peer-to-peer exchange.
- ▪ To encourage international teamwork and joint leadership between student coordinators in London and Somaliland and create opportunities for collaborative research in global mental healthcare.

Aqoon’s aims are achieved by pairing medical students from the UK and Somaliland for a set of ten fortnightly peer-to-peer global mental health e-learning discussion tutorials, using the instant messaging function of the website, MedicineAfrica. Students receive a set of ten suggested themes (Additional file 1: Appendix 1) for meetings but are free to deviate from these. They sign up to Terms of Reference (Additional file 1: Appendix 2) prior to participation, including agreement to anonymize details of clinical cases and complete evaluation questionnaires (Additional file 1: Appendix 3) before and after Aqoon.

The partnership is coordinated by one student at KCL and two at Hargeisa and Amoud Universities, Somaliland, known as Mental Health Reps, who sign up to terms of reference (Additional file 1: Appendix 4). Both are appointed from a pool of applicants by previous Aqoon coordinators and KSP volunteer staff. Both coordinators publicize Aqoon to students at their respective universities, predominantly via email in the UK and face-to-face contact in Somaliland. Once interested students are identified, they write a short introduction letter to their partner accompanied by a photograph, describing themselves and their reasons for participating. This feature was added to discourage participants from dropping out early or making little effort to contact their partner, by personalizing the link from the outset. Students are not usually matched using specific criteria, but it is recommended that students are matched by their level of mental health teaching or clinical exposure, to equalize partners’ baseline knowledge as far as possible.

Students are encouraged to meet weekly or fortnightly, with regular emails from their local coordinator reminding them to meet and complete brief questionnaires. Problems may arise with students making initial contact and finding mutually suitable times to meet, taking time-zone variation, cultural differences and computer and internet access into account. Local coordinators act as mediators to overcome these barriers, when required. Upon completion of ten sessions, participants complete a final online questionnaire and receive a certificate of participation for their educational portfolios. Coordinators endeavor to ensure that meetings begin sufficiently early in the academic year that they are completed prior to study leave for end-of-year examinations.

A new branch of Aqoon was piloted, linking KCL nursing students with peers in Somaliland, named *Sahan*, meaning *pioneer* or *adventurer*. A central challenge was facilitating Somaliland students to log onto the MedicineAfrica website at the same time as their partner in the UK, but English language levels have not been a barrier to productive communication in either program.

It can be challenging to devise a feasible and suitable evaluation method to meaningfully assess whether the aims of the Aqoon model are met. The questionnaire website, SurveyMonkey.com was found to be an accessible means of eliciting feedback from participants in both the UK and Somaliland. In the first 2 years of Aqoon, students were asked to complete a pre- and post-Aqoon questionnaire as well as a post-meeting questionnaire after each session. Participant feedback indicated that this approach was excessively demanding and in subsequent iterations, a single pre- and post-Aqoon survey was used. In the pilot phase, exclusively qualitative feedback was sought, about participants’ baseline views and objectives, about each session’s level of enjoyment, academic helpfulness and interest and, afterwards, students’ views on what was discussed, learned, gained and suggested improvements to the program. In subsequent iterations, more quantitative responses were sought (Additional file 1: Appendix 3), using the validated Attitudes Toward Psychiatry (ATP 30) questionnaire [24], which assesses levels of positivity and stigma towards the speciality. This enabled median ATP 30 scores to be compared pre- and post-participation using Wilcoxon signed rank tests. Formal assessment of the experiences and views of the UK and Somaliland coordinators were not sought, but this is recommended for future programs. Informal views are presented here.

## Results

An initial pilot of the Aqoon model pairing 10 UK and 10 Somaliland students found positive results [25]. In the follow-up partnership with a cohort of 24 UK and 24 Somaliland students, 98 questionnaires were completed after individual meetings: 41 by Somaliland students and 57 by KCL students. Four pairs (17%) reported meeting a full ten times but it was not possible to determine the total number of meetings held, because sessions not reported in questionnaires were not logged by the MedicineAfrica website. 18 UK and 14 Somaliland students completed post-Aqoon surveys (75% and 58% response rate, respectively) [26]. The specific content of participants’ discussions was not examined, to encourage frank discussion and debate between students, but qualitative feedback indicated that the topic suggestions provided (Additional file 1: Appendix 1) were positively received as offering a structure around which discussions of broader themes could develop. Over time, it has been felt by coordinators that asking students to commit to a minimum of eight sessions is the most feasible request that affords sufficient participation in the Aqoon model. Actively investigating how many sessions are required by measuring student knowledge and attitudes for a hypothesised ‘dose-response’ effect is one interesting potential research question.

Quantitative findings demonstrated that enjoyment, interest, and academic helpfulness were rated highly by students in Somaliland and moderately by students in the UK. Qualitative findings identified more gains in factual knowledge for Somaliland students, whereas UK students reported more cross-cultural learning.

Initial attitudes to psychiatry and responses to stigma questions were not significantly different between KCL and Somaliland students. Somaliland students’ scores on the ATP 30 questionnaire were significantly more positive post-participation, whereas UK students’ attitudes remained stable. While responses to most questions assessing stigmatized beliefs remained stable in both groups, Somaliland students’ responses were less stigmatized with respect to believing that people with mental illness can make decisions about their treatment, after participating in Aqoon. The proportion who would consider a career in psychiatry was stable among KCL students but increased from 55% to 93% among Somaliland students.

### Barriers to Partnership

After participating in Aqoon, students were asked, **‘**If you did not complete Aqoon, please tell us what problems you encountered and how you think we can address them**’**. 16 students (9 from KCL and 7 from Somaliland Universities) described reasons for not completing the full ten sessions, which were indexed into themes using content analysis. The commonest reasons for non-completion of the partnership among both groups were difficulty finding mutually suitable times, internet or website difficulties, clashing deadlines or examination times and difficulty communicating to plan meetings.

Two Somaliland students clarified that, out of politeness, they did not assert their own availability or seek help from the coordinators to reconnect with their partner, highlighting the need to empower mutual decision-making:

“Main problem was time of coming online. My partner used to select time which is suitable and I used to respect it. My suggestion is to persuade every participant to respect chances and opinions of his or her partner.”

“I used to remind regularly by email & I declined to push her through the coordinators… the best way of addressing this issue is to make sure whether those students registered to the program is interested to start and finish.”

However, students stressed the benefits of participation in spite of challenges:

“I have not ‘officially’ completed the sessions as my partner at times has not had any internet access and more recently is in the middle of exams. But we both have gotten so much from the few meetings that we have had [that we] plan on completing the remaining sessions as soon as possible keeping in contact even after this project officially ends.”

### Improvements

After completing Aqoon, participants were asked, ‘Please tell us three things which we should improve in the Aqoon project’ and their answers were indexed into themes using content analysis.

KCL and Somaliland students’ responses were broadly similar. The commonest suggestions from both groups were expansions of the model to include additional sessions, specialties, meetings or formats, better motivation of students to participate and strictly arranged meeting times. These comments suggest that the partnership was favorably received by students, but that logistics of meeting remained a central challenge.

### Evaluation

After Aqoon, all 14 Somaliland students and 17 out of 18 KCL respondents said they would recommend Aqoon to a friend, with one ‘maybe’. Students’ evaluations were positive even where the partnership was not completed:

“This was a very good project, a great chance to make a friend in a different continent and to really understand how psychiatry works differently in different cultures but also to question the way that psychiatric patients are approached and treated here and to analyze their care in detail. I learnt a lot from this project and really felt that it was of great benefit and would without a question recommend it to a friend.”

“The Aqoon project is a very unique and valuable experience and should definitely be undertaken by everyone. I have realized how views about a particular subject can differ not only between two people but two different cultures and I can now appreciate why this may be and also have learnt how to approach people especially when asking about sensitive topics. I feel that now when I take a psychiatry history, I am more sensitive and pay more attention to how I go about asking certain questions. This project has helped with such a vital clinical skill I feel I may not have otherwise noticed.”

“The Aqoon project was great as it allowed me to really think about psychiatry in a relaxed context and in an African context. It was great to have the weekly topics and then by the end to talk about what we were interested in. At first my partner would only talk about the specific guidelines given but eventually felt freer to discuss things not directly related.”

An important benefit in terms of leadership is that Aqoon enabled medical student coordinators in both countries to participate in collaborative research, which yielded academic presentations and publications. Better support for research from within low-income countries has been central to recent calls for improved global mental health [27].

### Lessons Learned

In 1 year, problems with the MedicineAfrica website delayed the beginning of Aqoon, meaning few participants were able to complete ten sessions before examinations began. Furthermore, communication difficulties between coordinators from the UK and Somaliland inhibited progress, compounded by a lack of specificity in their terms of reference (Additional file 1: Appendix 4), yielding a number of lessons:

- ▪ Strong leadership is required from coordinators on both sides of a student global health link.
- ▪ Coordinators in both countries need a good working relationship, grounded in previous collaboration or partnership-working, where possible.
- ▪ Coordinators require swift, efficient communication channels through which to mediate when student pairs struggle to find mutually suitable meeting times.
- ▪ Division of labor between coordinators should be as equal as possible, to ensure mutual contribution to the partnership and avoid hierarchical imbalance.
- ▪ Coordinators require senior support in their own country from a clinician or academic with experience of the partnership, to assist with difficult situations and provide guidance and leadership for research and evaluation. Seniors may need to mediate in the case of communication breakdown between coordinators.
- ▪ Partnerships require University-level support, through student unions or student societies, such as KCL Psychiatry Society, a network of medical students to whom Aqoon was advertised.

A coordinator who encountered communication difficulties with their overseas counterpart commented that:

“A pre-existing bond between coordinators is an important aspect of the program’s well-being. Future cycles should perhaps look to recruit coordinators who took part in the Aqoon program previously as this presupposes a certain level of interest in the program. Once recruited, coordinators should spend time getting to know each other through Skype meetings as opposed to just faceless e-mail communication, and should make aims to then also perhaps have fortnightly Skype meetings to discuss progress and problems.”

In addition to these lessons in leadership of peer-to-peer e-learning partnerships, several further considerations emerged from our experiences in Somaliland. Central to Aqoon is the equal relationship between students in both countries: there is no suggestion of hierarchy between participants, considering themselves mutual participants in shared learning.

While English language was not, in general, a challenge to participation by Somaliland students, the importance of clear, concise email communication by UK-based coordinators became apparent. It may be tempting to condense large amounts of information for participants or fellow coordinators into a single email, but the importance of brief, clear paragraphs with obvious points for response may need to be emphasized.

Some participants were disappointed by Aqoon because they expected it to be more directly applicable to examination revision. However, when final evaluations were analyzed, the cross-cultural and psychosocial aspects of global mental health learning were most valued by students in both countries: learning which cannot be derived from other educational sources. It is therefore important in initial advertising, information evenings and correspondence, to ensure that prospective participants have realistic expectations of the purpose and aims of peer-to-peer global health education partnerships. Suggested themes and prompts for each meeting should also be adapted to draw students away from basic revision of clinical facts towards discussion and debate around grey areas and ethical challenges within global health. The use of polemical images and articles as prompts is one suggested strategy. Another involves structuring the content of online e-learning sessions around a curriculum such as the World Health Organization mhGAP Intervention Guide [28].

Many of the challenges of managing student meetings entailed managing frustrations on both sides regarding partners’ inflexibility in arranging mutually suitable meeting times or lack of reliability. This resulted from limited insight from KCL students into the logistical challenges facing Somaliland students in accessing computers with reliable internet access and cultural barriers preventing Somaliland students from voicing their needs. An information sheet about the cultural and logistical context of each country is recommended, to ensure that participating students negotiate their e-learning sessions with a more contextualized appreciation of logistical circumstances. Furthermore, participants should be encouraged to devote time in their first meeting to discussing the practicalities of the e-learning partnership and identifying any barriers which might affect their availability in future, to ensure understanding and transparency from the outset.

Peer-to-peer online learning in groups was suggested by one participating student as a means to overcome difficulties associated with pairing. However, this has been resisted in the Aqoon context, because of positive ratings from students of the personal nature of peer learning with a single partner and the regular reporting of friendship as a key gain from participation, which would be limited by focusing on groups.

## Discussion

This article seeks to engage in the dissemination of learning from successful partnerships advocated by AMPATH, to inform the design of alternative models around the world. Crucial to the success and progress of Aqoon has been tailoring its design to the educational requirements and logistical realities in Somaliland and the UK, and flexibility in adapting the model to reflect on-going feedback and evaluation. This article cannot advocate identical replication of the Aqoon model in other paired institutional partnerships without initial assessment of contextual needs and practicalities. Rather, Aqoon is here presented as a novel approach to a specific set of learning needs, to illustrate the dynamic process by which a peer-to-peer e-learning partnership for global health education may be designed, piloted, evaluated, modified and re-tested.

In particular, the modality through which peer-to-peer e-learning takes place must be directed by the logistical restrictions of the low-income setting’s resources. The instant messaging format of MedicineAfrica is ideally suited to Somaliland, where generally reliable internet is available, at a low bandwidth. In other countries, internet access is too unreliable to justify immediate-response learning formats and another modality is recommended, such as correspondence via extended weekly email exchanges, or a group blog [29], in which students post longer comments at a time convenient to them.

All of the four obstacles to successful electronic partnership identified by AMPATH, ‘cultural differences and geographic distances’, ‘miscommunication and misunderstanding’, ‘maintaining funding and momentum for initiatives’ and ‘lack of consistent, basic services such as electricity and internet connectivity’ were pertinent to Aqoon. Cultural and geographical differences enhanced the attractiveness and perceived value of participation, but contributed to challenges of engagement. Miscommunication and misunderstanding were key factors in failures to meet online and although Aqoon was cost-neutral, maintaining the momentum to complete all sessions was a central challenge, especially as competing commitments such as examinations approached. Access to internet connectivity was in part addressed by the low bandwidth site, MedicineAfrica, but as access to smart phones develops and internet quality improves, the potential to adapt Aqoon to an App or other mobile modality will become increasingly viable.

## Conclusions

There is a growing emphasis on global health in medical education, despite cuts in university funding in high income countries and restricted educational resources in low-income countries. Peer education initiatives using telemedicine are increasingly popular but not always well-studied. Aqoon, a novel peer-to-peer global mental health e-learning partnership between the UK and Somaliland building upon a wider institutional partnership, is one innovative approach, which solely employs existing resources. This model proved popular when expanded to a larger cohort, yielding lessons learned through its development and evaluation. Challenges including communication difficulties and balancing participants’ expectations prompted key recommendations for institutional partners considering their own peer-to-peer program, in particular the need to emphasize commitment from the outset. Aqoon illustrates the potential benefits to medical students in dramatically different locations of online peer-to-peer education and expands the scope of a paired institutional partnership beyond qualified clinicians, to the healthcare professionals of the future.

## Additional file

Additional file 1: Appendix 1. Aqoon Discussion Topics. **Appendix 2.** Aqoon Medical Student Terms of Reference. **Appendix 3.** SurveyMonkey.com Post-Aqoon Evaluation Questionnaire. **Appendix 4.** Terms of Reference for KSP Mental Health Reps. (DOCX 752 kb)

## Abbreviations

AMPATH: Academic Model Providing Access to Healthcare
KCL: King’s College London
KSP: King’s Somaliland Partnership
THET: Tropical Health and Education Trust
UK: United Kingdom
WHO: World Health Organization

## References

[1] Jones A (2016). Envisioning a global health partnership movement. Glob Health.

[2] United Nations: United Nations millennium declaration. 2000. http://www.un.org/millennium/declaration/ares552e.pdf. Accessed 30 April 2015.

[3] United Nations: Sustainable development goals. 2015. https://sustainabledevelopment.un.org/?menu=1300. Accessed 16 March 2016.

[4] Tierney WM, Kanter AS, Fraser HSF, Bailey C (2010). A toolkit for e-health partnerships in low-income nations. Health Affair.

[5] Mackinnon R, Haque A, Stark P (2009). Peer teaching: by students for students. A student-led initiative. Clin Teach.

[6] Harden RM, Hart IR (2002). An international virtual medical school (IVIMEDS): The future for medical education?. Med Teach.

[7] Evans P, Suzuki Y, Begg M, Lam W (2008). Can medical students from two cultures learn effectively from a shared web-based learning environment?. Med Educ.

[8] Cook DA, Garside S, Levinson AJ, Dupras DM, Montori VM (2010). What do we mean by web-based learning? A systematic review of the variability of interventions. Med Educ.

[9] Ravenscroft A, Tait K, Hughes I (1998). Beyond the media: Knowledge level interaction and guided integration for CBL systems. Comput Educ.

[10] Fletcher-Flinn C, Gravatt B (1995). The efficacy of computer assisted instruction (CAI): A meta-analysis of outcomes. J Educ Comput Res.

[11] Thakore H, McMahon T (2006). Virtually there: E-learning in medical education. Clin Teach.

[12] Kerfoot BP, Conlin PR, McMahon GT (2006). Comparison of delivery modes for online medical education. Med Educ.

[13] General Medical Council: Tomorrow’s Doctors. 2009. http://www.gmc-uk.org/Tomorrow_s_Doctors_1214.pdf_48905759.pdf. Accessed 30 April 2015.

[14] Higher Education Funding Council for England: Recurrent grants for 2011-12. 2011. http://www.hefce.ac.uk/pubs/year/2011/201107. Accessed 30 April 2015.

[15] Jarabi BO. Review of various population estimates for Somaliland, Puntland and South-Central Somalia. Independent Consultancy Mission. 2007. https://scholar.google.co.uk/scholar?cluster=6514650994262477955&hl=en&as_sdt=2005&sciodt=0,5.

[16] Syed Sheriff RJ, Baraco AFH, Nour A, Warsame AM, Peachey K, Haibe F (2010). Improving human resource provision for mental health in Somaliland. Psychiat Serv.

[17] Syed Sheriff RJ, Reggi M, Mohamed A, Haibe F, Whitwell S, Jenkins R (2011). Mental health in Somalia. Int Psychiat.

[18] Syed Sheriff RJ, Leather AJM, Stanton E, Lemer C, Mountford J (2009). Leadership and global health. Clinical leadership: bridging the divide.

[19] Leather AJM, Butterfield C, Peachey K, Silverman M, Syed Sheriff R (2010). International health links movement expands in the United Kingdom. Int Health.

[20] Leather A, Ismail EA, Ali R, Abdi YA, Abby MH, Gulaid SA (2006). Working together to rebuild healthcare in post-conflict Somaliland. Lancet.

[21] Syed Sheriff RJ, Bass N, Hughes P, Ade-Odunlade P, Ismail A, Whitwell S (2013). Use of interactive teaching techniques to introduce mental health training to medical schools in a resource poor setting. Afr J Psychiat.

[22] Syed Sheriff RJ, Whitwell S (2012). An innovative approach to integrating mental health into health systems: strengthening activities in Somaliland. Interv Int J Ment Health Psychosoc Work Couns Areas Armed Conflict.

[23] Finlayson AET, Baraco A, Cronin N, Johnson O, Little S, Nuur A (2010). An international, case-based, distance-learning collaboration between the UK and Somaliland using a real-time clinical education website. J Telemed Telecare.

[24] Burra P, Kalin R, Leichner P, Waldron JJ, Handforth JR, Jarrett FJ (2009). The ATP 30 – A scale for measuring medical students’ attitudes to psychiatry. Med Educ.

[25] Keynejad R, Ali FR, Finlayson AET, Handuleh J, Adem G, Bowen JST (2013). Telemedicine for peer-to-peer psychiatry learning between U.K. and Somaliland medical students. Acad Psychiatr.

[26] Keynejad R, Garratt E, Adem G, Finlayson A, Whitwell S, Sheriff RS. Improved attitudes to psychiatry: a global mental health peer-to-peer e-learning partnership. Academic Psychiatry. 2014:1–8. 10.1007/s40596-014-0206-8 25124879

[27] Collins PY, Patel V, Joestl SS (2011). Grand challenges in global mental health. Nature.

[28] World Health Organization: mhGAP Intervention Guide. 2010. http://whqlibdoc.who.int/publications/2010/9789241548069_eng.pdf?ua=1. Accessed 30 April 2015.

[29] Ladyshewsky RK, Gardner P (2008). Peer assisted learning and blogging: A strategy to promote reflective practice during clinical fieldwork. Australas J Educ Technol.

