# Supplementary material for: Global health partnership for student peer-to-peer psychiatry e-learning: Lessons learned
Source: Global Health. 2016 Dec 3;12:82. doi: 10.1186/s12992-016-0221-5 (PMC5135829; doi:10.1186/s12992-016-0221-5)
Supplement: Additional file 1: Appendix 1. — Aqoon Discussion Topics. Appendix 2. Aqoon Medical Student Terms of Reference. Appendix 3. SurveyMonkey.com Post-Aqoon Evaluation Questionnaire. Appendix 4. Terms of Reference for KSP Mental Health Reps. (DOCX 752 kb) [file 12992_2016_221_MOESM1_ESM.docx]

Appendices

Appendix 1: Aqoon Discussion Topics

This is a set of themes for each of the 10 sessions in which you will be meeting up with your partner through Medicine Africa. In advance, you must agree a date and time with your partner and set up a “meeting” on the website, which will enable you to communicate through instant messaging.

The themes are by no means set in stone and if you want to move outside of these you are very welcome to. However, from experience we have found that students appreciate having a framework to guide their meetings, so that you can prepare and read up a bit on that topic before your meeting.

It is very important that after every single meeting you complete our very short questionnaire: <http://www.surveymonkey.com/s/FP6SS2F>

It will take a maximum of 2 minutes to complete and will ensure that we can evaluate the partnership, to decide if it will run next year, and if so, what improvements need to be made.

Many thanks and enjoy!

Session 1: Week of 17^th^ January: Getting to know each other

- You and your family
- Why you are studying Medicine
- Why you wanted a partner in Somaliland/London
- Your interests and hobbies
- What you’d like to get out of the partnership
- What you think about psychiatry – what is it? What types of treatment can it offer?
- What is mental health? What is mental illness?
- What types of psychiatric illness have you seen in patients (remember to anonymize any patient identifiers like names)?

Session 2: Week of 31st January: Cultural perspectives and Stigma

- Does medication help people who are mentally unwell?
- Does talking help people who have a mental health problem?
- How are people with mental illness treated in society?
- How do you think people would react if you had a mental illness?
- Does your culture (country, religion, community) have its own way of explaining mental illness?
- What problems do people with mental health problems face which are not faced by people with physical health problems?
- Are employers understanding about mental health?

Session 3: Week of 14^th^ February: Affective disorders 1

- What is depression?
- Is it an illness? Can it be treated?
- Where does ordinary sadness stop and depression begin?

Session 4: Week of 28^th^ February: Affective disorders 2

- How do people in your community/culture react to people who harm themselves?
- What have you read about suicidal feelings? How do you assess a patient’s risk of suicide?
- What does it mean to be manic? What is bipolar disorder? How is it treated?

Session 5: Week of 14^th^ March: Anxiety

- What is ‘normal’ and ‘abnormal’ anxiety?
- Have you encountered any patients with anxiety (remember to anonymise any identifying features)?
- How can they be helped?
- Have you met any patients with problems of obsession or compulsion?
- What did you learn from them?

Session 6: Week of 28^th^ March: Substance misuse

- What is Khat and what is cannabis?
- How are they used in your culture/community and how do they affect mental health?
- What social problems come out of these two substances?
- Are they the main substance problem you have noticed?
- What other drugs cause patients problems? Alcohol, stimulants, opiates, cigarettes.
- What can psychiatry do to help people with substance misuse problems?

Session 7: Week of 11^th^ April: Psychosis

- What is psychosis?
- Have you met any patients with psychosis? (Remember to anonymise identifiers)
- Have you seen any films or read any books about it?
- How are people who are psychotic viewed by your culture/community?
- What can psychiatry do to help people with psychosis?
- What are the reasons that people develop psychosis?

Session 8: Week of 25^th^ April: PTSD

- What is post traumatic stress disorder?
- Is it a disease/disorder?
- Have you met any patients with this condition, or read about it or seen films about it?
- What can psychiatry do to help people with PTSD?
- What other disorders may overlap with PTSD – what are the differential diagnoses?

Session 9: Week of 9^th^ May: A topic in which you are both interested

Talk about why this psychiatric topic interests you and what you know about it. Discuss the symptoms, differential diagnoses, treatment options and challenges for the doctor and the patient.

Session 10: Week of 23^rd^ May: Final Session

A topic of your choice which you are both interested in. Talk about why that psychiatric topic interests you and what you know about it. Discuss the symptoms, differential diagnoses, treatment options and challenges for the doctor and the patient.

Finally, discuss what you have gained from the partnership and what your future plans are. Which area of medicine do you think you will specialise in and why? In what ways will your knowledge of psychiatry help you in this field? What areas of overlap are there? How do you progress to becoming a doctor in Somaliland/UK? Discuss if/how you and your partner will keep in touch from now on. You are very welcome to carry on meeting formally or informally, or to move to email if you find that a more convenient correspondence.

Don’t forget to fill in an evaluation after each session! <http://www.surveymonkey.com/s/FP6SS2F>

Appendix 2: Aqoon Medical Student Terms of Reference


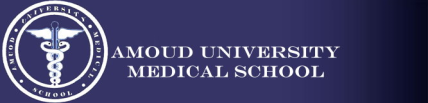

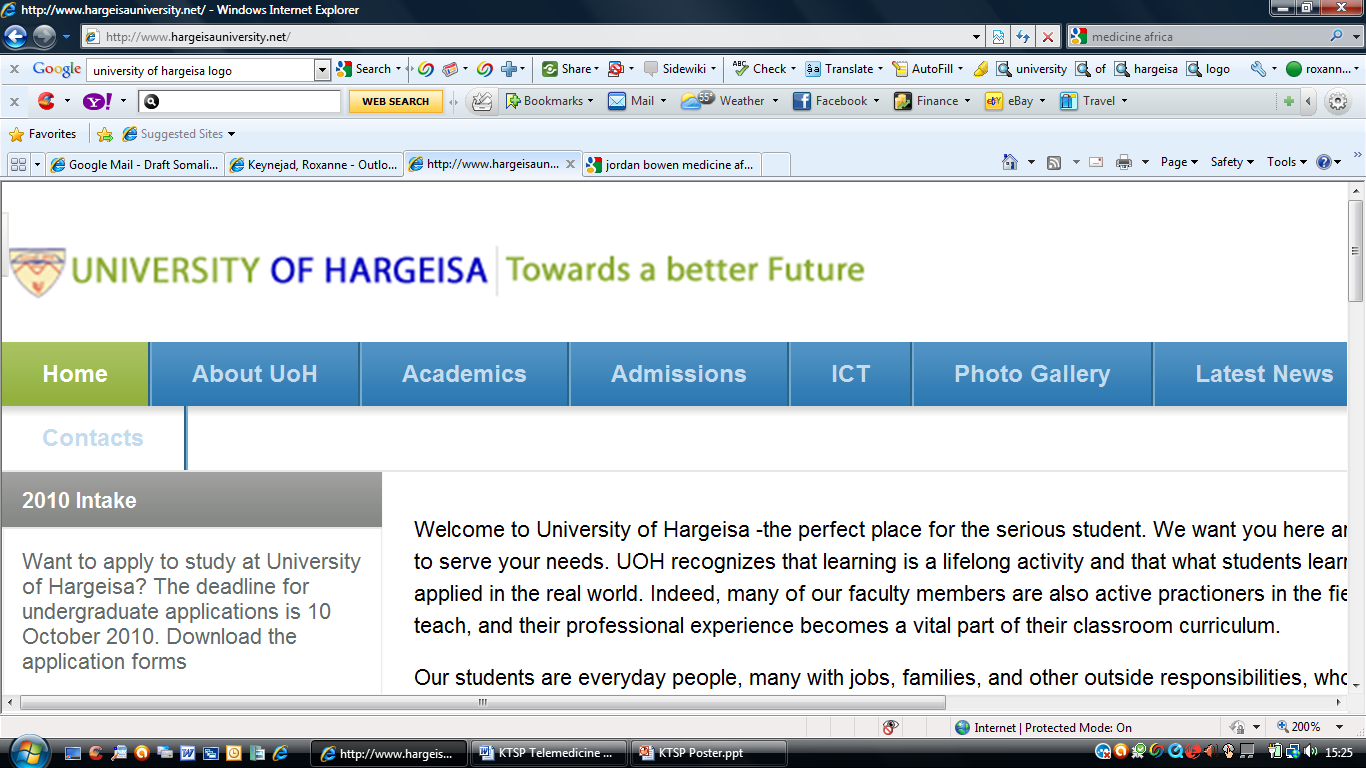

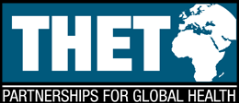

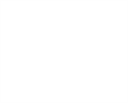


KCL Psychiatry Society, in collaboration with King’s Somaliland Partnership is launching the second year of a link which partners medical students at King’s College London with an interest in psychiatry with medical students at Amoud University and Hargeisa University, Somaliland, with a similar interest.

Terms of Reference:

1. Medical students will be required to commit one hour of their time, every two weeks, to communicating with their partner student in Somaliland or the UK via the website, Medicine Africa.
2. If either medical student is unable to attend one of these sessions, they must inform their partner abroad at least 48 hours in advance, via email, and CC both the KCL Psychiatry Society rep and the Somaliland mental health rep into the email, clearly stating when they will reschedule this session.
3. Every one hour meeting session will have a focus for discussion, beginning with getting to know each other, each other’s interests and what it is like studying Medicine for them. This will progress to specific topics in psychiatry such as depression, drugs and mental health (comparing khat to cannabis), psychosis and so on.
4. The objective of this partnership is for medical students to learn about cross-cultural psychiatry: the similarities across the globe and the location-specific challenges faced by clinicians in the UK and in Somaliland.
5. This scheme is a mutually beneficial exchange between medical students: there is no level at which one side takes a teaching role and the other a student role.
6. The pilot will run from January to May 2011, with students meeting for ten one hour meetings.
7. Confidentiality of any patient information that is shared by medical students is very important. Students must abide by the code of confidentiality set out by their medical school and must anonymise any patient-identifying details when discussing cases of interest.
8. Any problems or complaints will be addressed by the mental health reps in Somaliland and the KCL rep: [kclsomaliland@gmail.com](mailto:kclsomaliland@gmail.com) in the UK.
9. Students will not be permitted to request or offer donations of money or other items to support each other’s education. This is because this type of arrangement sets a precedent for future years in which new students would be expected to donate: an unsustainable setup. This is not the objective of the KSP medical student partnership.
10. Students completing all ten meetings of the project and completing all questionnaires will receive a certificate from the Institute of Psychiatry.
11. Students taking part must agree to complete a very short questionnaire at the end of each one hour meeting to enable us to evaluate the partnership at the end of the pilot, via the SurveyMonkey website, in addition to one short questionnaire at the start and one at the end of the partnership.

All participating medical students must sign up to agree to these terms of reference.

Appendix 3: SurveyMonkey.com Post-Aqoon Evaluation Questionnaire


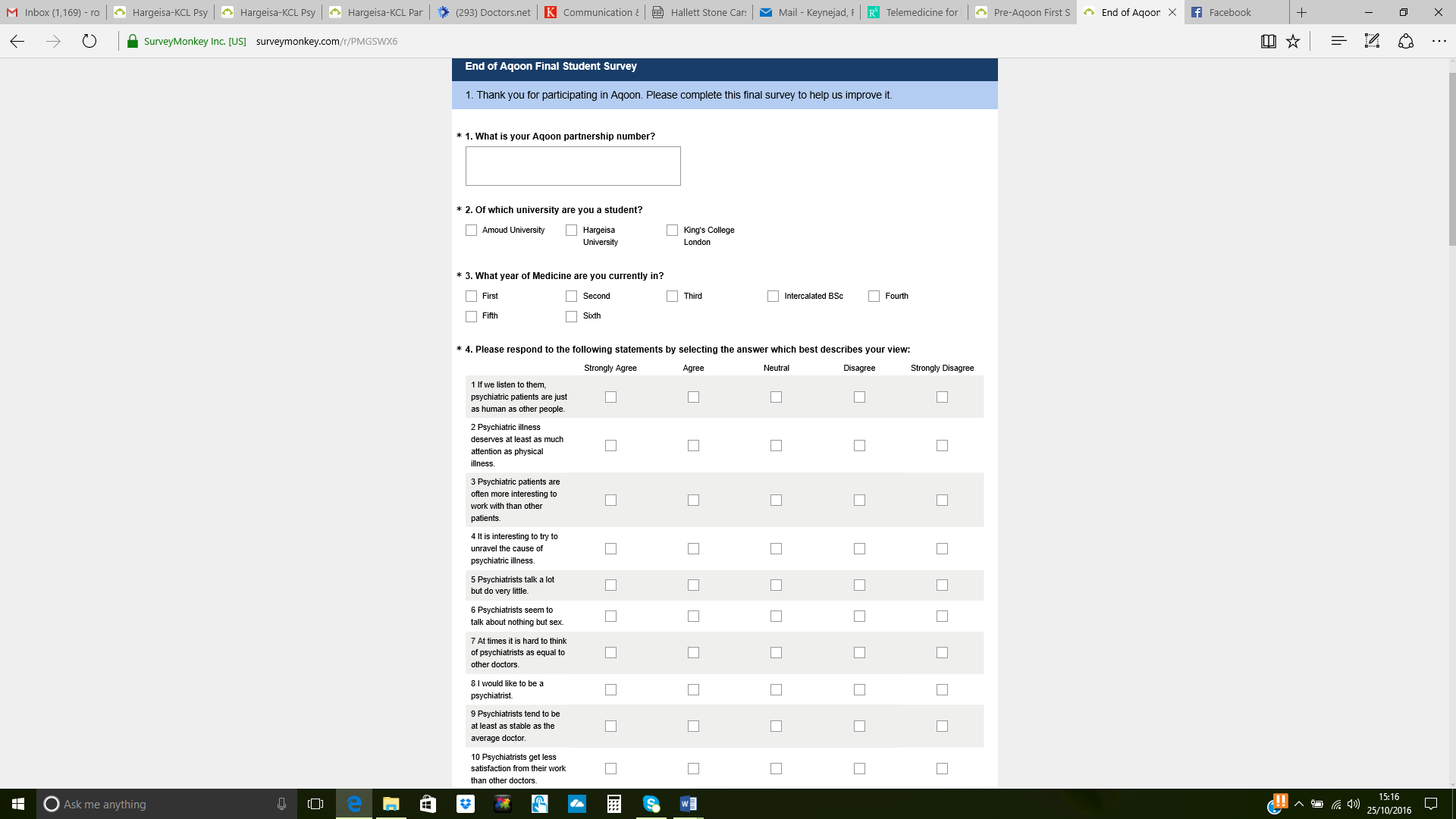

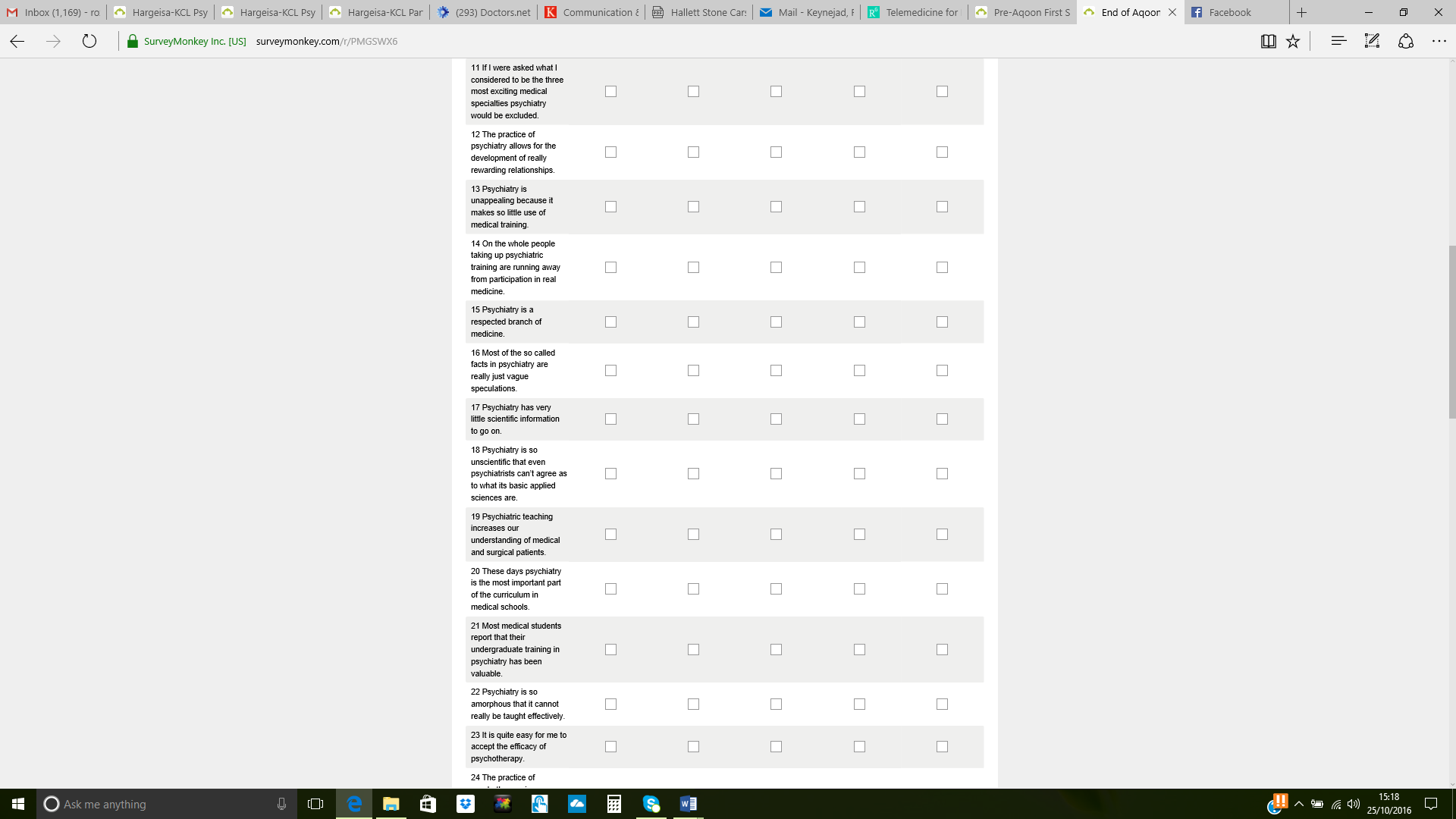

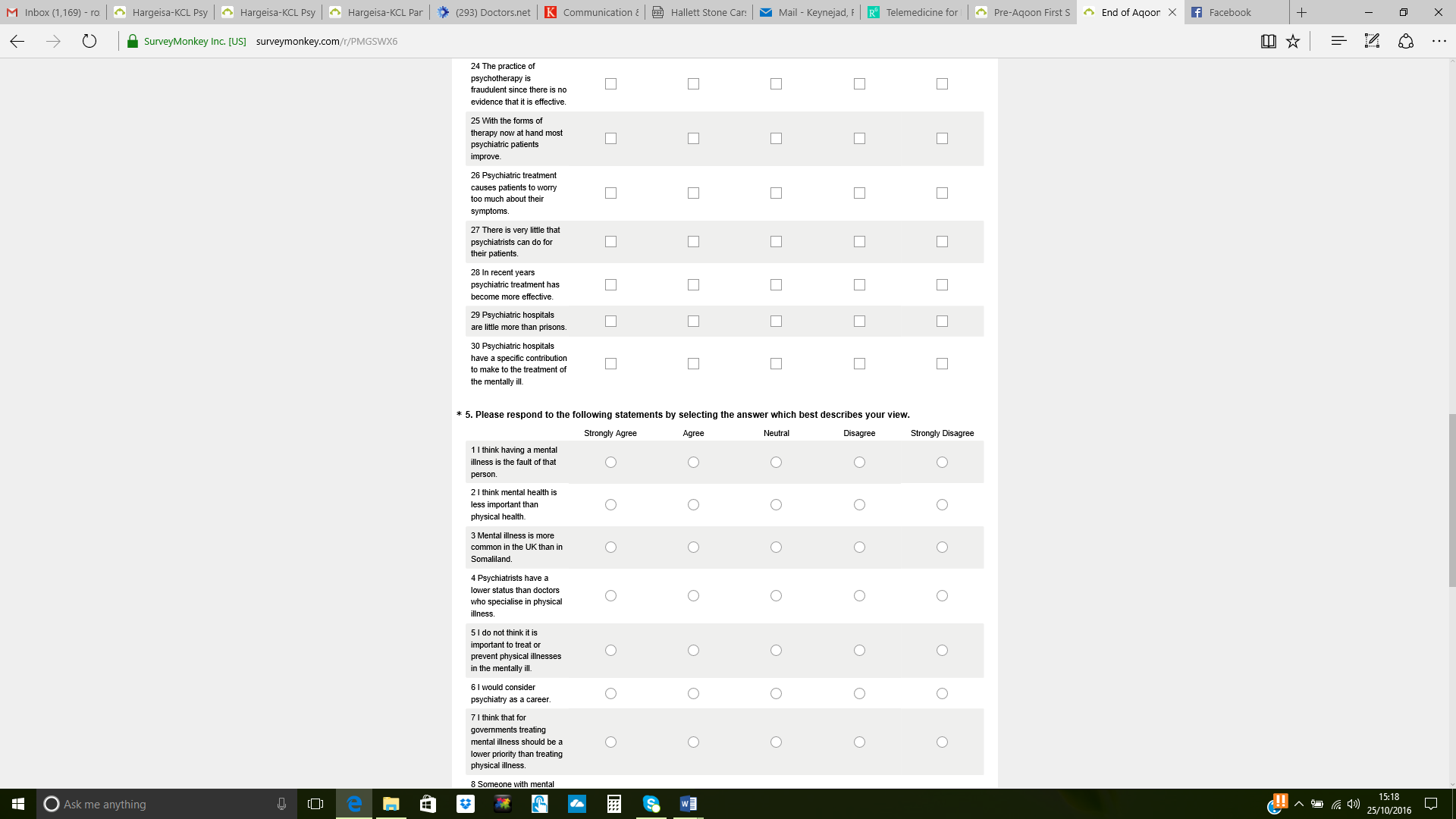

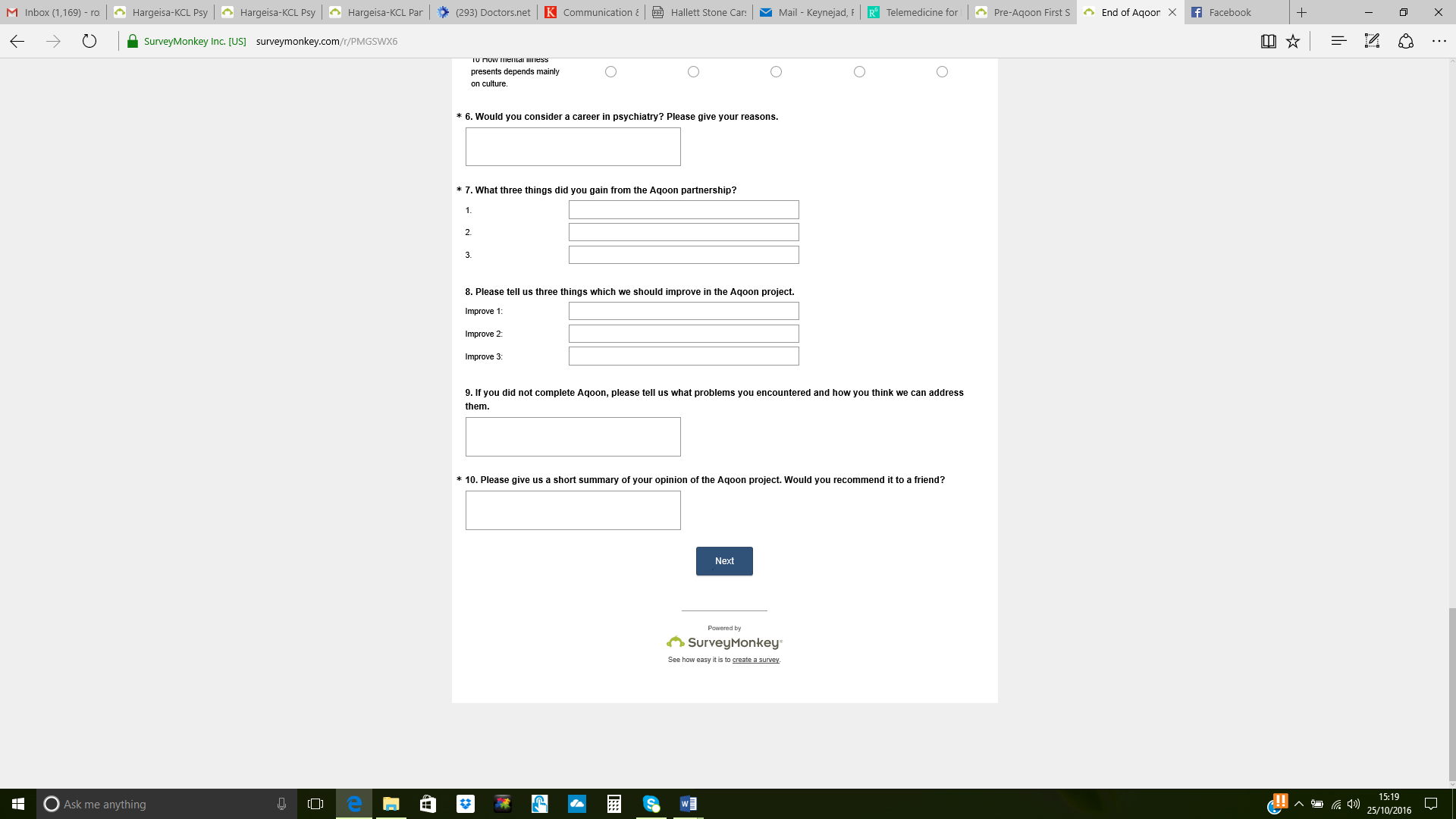


Appendix 4: Terms of Reference for KSP Mental Health Reps

King’s Somaliland Partnership (KSP) Mental Health Reps are Somaliland doctors at intern level with a particular aptitude for mental health. They are chosen for their skill and enthusiasm in the specialty of mental health and play an important role in developing mental health teaching and practice in Somaliland.
The role is a **one year voluntary post without financial remuneration** or accommodation and is recognized by the Deans of Amoud and Hargeisa Universities, KSP and the internship program as a position carrying responsibility and prestige. Mental Health Reps take on a leadership role, working closely with the KSP Mental Health Group to promote and sustain mental health in Somaliland. Their duties are:

**1. Teaching**

Mental Health Reps play a prominent role in the undergraduate mental health teaching course for Amoud and Hargeisa Universities. Mental Health Reps will have a key role with providing educational support and coordination with MedicineAfrica.com and other platforms. During face-to-face courses, Mental Health Reps co-lead group discussions and have the opportunity to develop teaching skills and take on independent lecturing and teaching duties supervised by the KSP team.

**2. Examining**

Mental Health Reps take responsibility for coordinating final year examinations in psychiatry, under the supervision and support of the KSP team. This includes working with seniors to develop a Multiple Choice Question Examination, writing OSCE scenarios and participating in examining. Examinations are of an international standard and their preparation requires organization, academic skill and an eye for detail.

**3. Clinical leadership in mental health**

As interns adopt clinical roles on the Hargeisa Group Hospital mental health ward, the new Mental Health Outpatient clinic at Borama and Berbera Mental Hospital, Mental Health Reps have an increasing role in supporting intern colleagues and coordinating intern supervision sessions between Somaliland and the UK through mhGAP.

**4. Research**

Previous Mental Health Reps have contributed to academic papers published in international journals: academic evaluation of KSP remains an important focus.

**5. Aqoon**

Mental Health Reps provide support to medical student coordinators of Aqoon, the peer-to-peer global mental health e-learning partnership between Somaliland and UK medical students.

**6. Contact with UK Mental health leads.**

Support is provided by KSP Mental Health leads in UK and the Reps are expected to be in regular contact by e-mail, MedicineAfrica.com or other platforms.
